# Supplementary material for: The effect of cognitive behavioural therapy on pain and disability in chronic non-specific low back pain: An overview of systematic reviews
Source: PLoS One. 2025 Jun 17;20(6):e0325122. doi: 10.1371/journal.pone.0325122 (PMC12173195; doi:10.1371/journal.pone.0325122)
Supplement: S3 Supporting Information — (DOCX) [file pone.0325122.s003.docx]

**S3 Supporting Information. Search Strategy.**

Ovid MEDLINE(R) ALL <1946 to April 20, 2024>

1 exp Back Pain/ 44908

2 exp Low Back Pain/ 26588

3 ("back pain" or "low back pain" or "LBP" or "CLBP" or "chronic back pain" or "chronic low back pain").mp. [mp=title, book title, abstract, original title, name of substance word, subject heading word, floating sub-heading word, keyword heading word, organism supplementary concept word, protocol supplementary concept word, rare disease supplementary concept word, unique identifier, synonyms, population supplementary concept word, anatomy supplementary concept word] 77333

4 ("backache" or "back-ache" or "back ache" or "back disorder").mp. [mp=title, book title, abstract, original title, name of substance word, subject heading word, floating sub-heading word, keyword heading word, organism supplementary concept word, protocol supplementary concept word, rare disease supplementary concept word, unique identifier, synonyms, population supplementary concept word, anatomy supplementary concept word] 4153

5 ("spine" or "spinal" or "lumbosacral" or "lumbar spine").mp. [mp=title, book title, abstract, original title, name of substance word, subject heading word, floating sub-heading word, keyword heading word, organism supplementary concept word, protocol supplementary concept word, rare disease supplementary concept word, unique identifier, synonyms, population supplementary concept word, anatomy supplementary concept word] 542661

6 ("lumbago" or "dorsalgia").mp. [mp=title, book title, abstract, original title, name of substance word, subject heading word, floating sub-heading word, keyword heading word, organism supplementary concept word, protocol supplementary concept word, rare disease supplementary concept word, unique identifier, synonyms, population supplementary concept word, anatomy supplementary concept word] 1636

7 exp Cognitive Behavioral Therapy/ 36493

8 ("cognitive behavio* therap*" or "cognitive function* therap*" or "CBT" or "CFT").mp. [mp=title, book title, abstract, original title, name of substance word, subject heading word, floating sub-heading word, keyword heading word, organism supplementary concept word, protocol supplementary concept word, rare disease supplementary concept word, unique identifier, synonyms, population supplementary concept word, anatomy supplementary concept word] 45774

9 "cognitive therap*".mp. [mp=title, book title, abstract, original title, name of substance word, subject heading word, floating sub-heading word, keyword heading word, organism supplementary concept word, protocol supplementary concept word, rare disease supplementary concept word, unique identifier, synonyms, population supplementary concept word, anatomy supplementary concept word] 4130

10 (cognitive adj4 therap*).mp. [mp=title, book title, abstract, original title, name of substance word, subject heading word, floating sub-heading word, keyword heading word, organism supplementary concept word, protocol supplementary concept word, rare disease supplementary concept word, unique identifier, synonyms, population supplementary concept word, anatomy supplementary concept word] 46126

11 (cognitive adj4 intervention*).mp. [mp=title, book title, abstract, original title, name of substance word, subject heading word, floating sub-heading word, keyword heading word, organism supplementary concept word, protocol supplementary concept word, rare disease supplementary concept word, unique identifier, synonyms, population supplementary concept word, anatomy supplementary concept word] 11301

12 exp psychotherapy/ 218323

13 ("psychotherap*" or "psychological therap*").mp. [mp=title, book title, abstract, original title, name of substance word, subject heading word, floating sub-heading word, keyword heading word, organism supplementary concept word, protocol supplementary concept word, rare disease supplementary concept word, unique identifier, synonyms, population supplementary concept word, anatomy supplementary concept word] 102115

14 exp "Systematic Review"/ 231907

15 exp Meta-Analysis/ 183235

16 ("systematic review" or "meta-analys?s" or "meta analys?s").mp. [mp=title, book title, abstract, original title, name of substance word, subject heading word, floating sub-heading word, keyword heading word, organism supplementary concept word, protocol supplementary concept word, rare disease supplementary concept word, unique identifier, synonyms, population supplementary concept word, anatomy supplementary concept word] 453038

17 exp Adult/ 7940535

18 exp Adolescent/ or exp Child/ or exp Infant/ 3962125

19 1 or 2 or 3 or 4 or 5 or 6 591293

20 7 or 8 or 9 or 10 or 11 or 12 or 13 260011

21 14 or 15 or 16 453038

22 18 not 17 2137425

23 19 and 20 and 21 256

24 23 not 22 256

Embase <1974 to 2024 April 20>

1 exp backache/ 135867

2 exp low back pain/ 72033

3 ("back pain" or "low back pain" or "lbp" or "clbp" or "chronic back pain" or "chronic low back pain").mp. [mp=title, abstract, heading word, drug trade name, original title, device manufacturer, drug manufacturer, device trade name, keyword heading word, floating subheading word, candidate term word] 118384

4 ("backache" or "back-ache" or "back ache" or "back disorder").mp. [mp=title, abstract, heading word, drug trade name, original title, device manufacturer, drug manufacturer, device trade name, keyword heading word, floating subheading word, candidate term word] 67816

5 ("spine" or "spinal" or "lumbosacral" or "lumbar spine").mp. [mp=title, abstract, heading word, drug trade name, original title, device manufacturer, drug manufacturer, device trade name, keyword heading word, floating subheading word, candidate term word] 721640

6 ("lumbago" or "dorsalgia").mp. [mp=title, abstract, heading word, drug trade name, original title, device manufacturer, drug manufacturer, device trade name, keyword heading word, floating subheading word, candidate term word] 2085

7 exp cognitive therapy/ 73183

8 exp cognitive behavioral therapy/ 26703

9 ("cognitive behavio* therap*" or "cognitive function* therap*" or "CBT" or "CFT").mp. [mp=title, abstract, heading word, drug trade name, original title, device manufacturer, drug manufacturer, device trade name, keyword heading word, floating subheading word, candidate term word] 54810

10 "cognitive therap*".mp. 46268

11 (cognitive adj4 therap*).mp. [mp=title, abstract, heading word, drug trade name, original title, device manufacturer, drug manufacturer, device trade name, keyword heading word, floating subheading word, candidate term word] 82639

12 (cognitive adj4 intervention*).mp. [mp=title, abstract, heading word, drug trade name, original title, device manufacturer, drug manufacturer, device trade name, keyword heading word, floating subheading word, candidate term word] 15528

13 exp psychotherapy/ 293595

14 ("psychotherap*" or "psychological therap*").mp. [mp=title, abstract, heading word, drug trade name, original title, device manufacturer, drug manufacturer, device trade name, keyword heading word, floating subheading word, candidate term word] 127100

15 exp "systematic review"/ 444867

16 exp meta analysis/ 298649

17 ("systematic review" or "meta analys?s" or "meta-analys?s").mp. [mp=title, abstract, heading word, drug trade name, original title, device manufacturer, drug manufacturer, device trade name, keyword heading word, floating subheading word, candidate term word] 735254

18 exp adult/ 10866103

19 exp juvenile/ 4050076

20 1 or 2 or 3 or 4 or 5 or 6 822910

21 7 or 8 or 9 or 10 or 11 or 12 or 13 or 14 336237

22 15 or 16 or 17 735254

23 19 not 18 2586144

24 20 and 21 and 22 750

25 24 not 23 739

Cochrane

Search Name: CBT LBP umbrella 20/4 psychotherapy

Date Run: 20/04/2024 16:10:20

Comment: umbrella review CBT LBP Cochrane 20/4

psychotherapy

ID Search Hits

#1 MeSH descriptor: [Back Pain] explode all trees 7210

#2 MeSH descriptor: [Low Back Pain] explode all trees 5822

#3 "back pain" OR "low back pain" OR "LBP" OR "CLBP" OR "chronic back pain" or "chronic low back pain" 17912

#4 "back ache" OR "backache" OR "back-ache" OR "back disorder" 4641

#5 "spine" OR "spinal" OR "lumbar spine" OR "lumbosacral" 50398

#6 "lumbago" OR "dorsalgia" 486

#7 MeSH descriptor: [Cognitive Behavioral Therapy] explode all trees 12896

#8 "CBT" OR "CFT" 11318

#9 cognitive NEAR/4 therap* 28684

#10 cognitive near/4 intervention* 9616

#11 cognitive function* therap* 24005

#12 cognitive behavio* therap* 32048

#13 MeSH descriptor: [Psychotherapy] explode all trees 33287

#14 psychotherap* 20248

#15 psychological therap* 35103

#16 MeSH descriptor: [Systematic Review] explode all trees 426

#17 MeSH descriptor: [Systematic Reviews as Topic] explode all trees 112

#18 MeSH descriptor: [Meta-Analysis as Topic] explode all trees 1451

#19 "systematic review" 15457

#20 meta NEXT analys?s 26723

#21 meta-analys?s 26723

#22 MeSH descriptor: [Adult] explode all trees 584918

#23 MeSH descriptor: [Adolescent] explode all trees 125417

#24 MeSH descriptor: [Child] explode all trees 77902

#25 MeSH descriptor: [Infant] explode all trees 41680

#26 {OR #1-#6} 64417

#27 {OR #7-#15} 97777

#28 {OR #16-#21} 31129

#29 {OR #23-#25} 189470

#30 #29 NOT #22 82050

#31 {AND #26-#28} 790

#32 #31 NOT #30 701

APA PsycInfo <1806 to April Week 3 2024>

1 exp Back Pain/ 4553

2 ("back pain" or "low back pain" or "LBP" or "CLBP" or "chronic back pain" or "chronic low back pain").mp. [mp=title, abstract, heading word, table of contents, key concepts, original title, tests & measures, mesh word] 7494

3 ("backache" or "back-ache" or "back ache" or "back disorder").mp. [mp=title, abstract, heading word, table of contents, key concepts, original title, tests & measures, mesh word] 184

4 ("spine" or "spinal" or "lumbosacral" or "lumbar spine").mp. [mp=title, abstract, heading word, table of contents, key concepts, original title, tests & measures, mesh word] 42457

5 ("lumbago" or "dorsalgia").mp. [mp=title, abstract, heading word, table of contents, key concepts, original title, tests & measures, mesh word] 48

6 exp Cognitive Behavior Therapy/ 26876

7 exp Cognitive Therapy/ 14057

8 ("cognitive behavio* therap*" or "cognitive function* therap*" or "CBT" or "CFT").mp. [mp=title, abstract, heading word, table of contents, key concepts, original title, tests & measures, mesh word] 38910

9 "cognitive therap*".mp. [mp=title, abstract, heading word, table of contents, key concepts, original title, tests & measures, mesh word] 27470

10 (cognitive adj4 therap*).mp. [mp=title, abstract, heading word, table of contents, key concepts, original title, tests & measures, mesh word] 57396

11 (cognitive adj4 intervention*).mp. [mp=title, abstract, heading word, table of contents, key concepts, original title, tests & measures, mesh word] 12303

12 exp psychotherapy/ 221961

13 ("psychotherap*" or "psychological therap*").mp. [mp=title, abstract, heading word, table of contents, key concepts, original title, tests & measures, mesh word] 216300

14 exp "Systematic Review"/ 789

15 exp Meta Analysis/ 5371

16 ("systematic review" or "meta-analys?s" or "meta analys?s").mp. [mp=title, abstract, heading word, table of contents, key concepts, original title, tests & measures, mesh word] 77892

17 adulthood 18 yrs older.ag. 2223536

18 adolescence 13 17 yrs.ag. 497096

19 childhood birth 12 yrs.ag. 602337

20 1 or 2 or 3 or 4 or 5 48752

21 6 or 7 or 8 or 9 or 10 or 11 or 12 or 13 340804

22 14 or 15 or 16 77892

23 18 or 19 881596

24 23 not 17 554495

25 20 and 21 and 22 49

26 25 not 24 49
