# Supplementary material for: The effect of cognitive behavioural therapy on pain and disability in chronic non-specific low back pain: An overview of systematic reviews
Source: PLoS One. 2025 Jun 17;20(6):e0325122. doi: 10.1371/journal.pone.0325122 (PMC12173195; doi:10.1371/journal.pone.0325122)
Supplement: S4 Table — (DOCX) [file pone.0325122.s004.docx]

| **Primary Studies** | Bahnamiri | Devonshire | Du | Hajihasani | Henschke | Ho | Lopez-de-Uralde-Villanueva | Pardos-Gascon | Petrucci | Rihn | van Middelkoop | Yang | Jurak |
| --- | --- | --- | --- | --- | --- | --- | --- | --- | --- | --- | --- | --- | --- |
| Abdolghadery 2014 | X |  |  |  |  |  |  |  |  |  |  |  |  |
| Alaranta 1994 |  |  |  |  |  | X |  |  |  |  |  |  |  |
| Aliyu 2018 |  |  |  |  |  | X |  |  |  |  |  |  |  |
| Altmaier 1992 |  |  |  | X | X |  |  |  |  |  |  |  |  |
| Basler 1997 |  |  |  |  | X |  |  |  | X |  |  | X |  |
| Bendix 1998 |  |  |  |  |  | X |  |  |  |  |  |  |  |
| Bendix 2000 |  |  |  |  |  | X |  |  |  |  |  |  |  |
| Beth 2021 |  |  |  |  |  |  |  |  |  |  |  | X |  |
| Brox 2003 |  |  |  |  | X | X |  |  |  | X |  | X |  |
| Brox 2006 |  |  |  |  |  |  |  |  |  | X |  |  |  |
| Buhrman 2004 |  |  |  |  |  |  |  |  |  |  |  | X |  |
| Carpenter 2012 |  |  |  |  |  |  |  |  |  |  |  | X |  |
| Castro 2022 |  | X |  |  |  |  |  |  |  |  |  |  |  |
| Cherkin 2016 | X |  |  |  |  | X |  | X | X |  |  | X |  |
| Chiauzzi 2010 |  |  |  |  |  | X |  |  |  |  |  |  |  |
| Christiansen 2010 |  |  |  |  |  | X |  |  |  |  |  | X |  |
| Critchley 2007 |  |  |  |  |  |  | X |  |  |  |  |  |  |
| Darnall 2021 |  |  |  |  |  |  |  |  |  |  |  |  | X |
| Day 2019 | X |  |  |  |  |  |  | X | X |  |  |  |  |
| De Jong 2005 |  |  |  |  |  |  | X |  |  |  |  |  |  |
| De Sousa 2009 |  |  |  |  |  |  |  |  |  |  |  |  | X |
| Devasahayam 2014 |  |  |  |  |  |  |  |  |  |  |  |  | X |
| Fairbank 2005 |  |  |  |  | X | X |  |  |  | X |  |  |  |
| Friedrich 1998 |  |  |  |  | X |  |  |  |  |  |  |  |  |
| Friedrich 2005 |  |  |  | X |  |  |  |  |  |  |  |  |  |
| Frost 1998 |  |  |  |  |  | X |  |  |  |  |  |  |  |
| Gardner 2019 |  |  |  |  |  |  |  |  |  |  |  |  | X |
| Ghadyani 2017 |  |  |  |  |  | X |  |  |  |  |  |  |  |
| Glombiewski 2010 |  |  |  |  |  | X |  |  |  |  |  |  |  |
| Godfrey 2020 |  |  | X |  |  |  |  |  |  |  |  | X | X |
| Gould 2020 |  |  |  |  |  |  |  |  |  |  |  | X |  |
| Harris 2017 |  |  |  |  |  |  |  |  |  |  |  | X |  |
| Hellum 2011 |  |  |  |  |  |  |  |  |  | X |  |  |  |
| Johnson 2007 |  |  |  |  | X | X |  |  | X |  |  | X |  |
| Kaapa 2006 |  |  |  |  |  |  |  |  |  |  |  |  | X |
| Khan 2014 |  |  |  | X |  | X |  |  |  |  |  | X |  |
| Khan 2016 |  |  |  |  |  |  |  |  | X |  |  |  |  |
| Khodadad 2019 |  | X |  |  |  |  |  |  |  |  |  |  | X |
| Kole-Snijders 1999 |  |  |  |  | X |  |  |  |  |  | X |  |  |
| Lamb 2010 |  |  |  |  |  | X |  |  |  |  |  |  |  |
| Lambeek 2010 |  |  |  |  |  |  | X |  |  |  |  |  |  |
| Leeuw 2008 |  |  |  |  | X | X | X |  |  |  |  |  |  |
| Linden 2014 |  |  |  |  |  |  |  |  | X |  |  |  | X |
| Linton 2000 |  |  |  |  |  |  |  |  | X |  |  |  |  |
| Linton 2008 |  |  |  |  | X |  |  |  |  |  |  |  |  |
| Macedo 2012 |  |  |  |  |  |  | X |  |  |  |  |  |  |
| Monticone 2013 |  |  |  | X |  | X |  |  | X |  |  | X | X |
| Monticone 2014 |  |  |  |  |  |  |  |  |  |  |  |  | X |
| Monticone 2016 |  |  |  |  |  | X |  |  |  |  |  |  | X |
| Newton-John 1995 |  |  |  |  | X |  |  |  |  |  | X | X |  |
| Nicholas 1991 |  |  |  | X | X | X |  |  |  |  |  |  | X |
| Nicholas 1992 |  |  |  | X | X |  |  |  |  |  |  |  |  |
| O'Keeffe 2020 |  | X |  |  |  | X |  |  |  |  |  |  | X |
| Petrozzi 2019 |  |  |  |  |  | X |  |  |  |  |  | X | X |
| Pincus 2015 |  |  |  |  |  |  |  |  | X |  |  | X |  |
| Reiner 2018 | X |  |  |  |  |  |  |  |  |  |  |  |  |
| Reme 2016 |  |  |  |  |  |  |  |  |  |  |  | X |  |
| Rose 1997 |  |  |  |  | X | X |  |  |  |  | X |  |  |
| Rutledge 2018 (a) |  |  |  |  |  |  |  |  | X |  |  | X |  |
| Rutledge 2018 (b) |  |  |  |  |  |  |  |  | X |  |  | X |  |
| Santaella da Fonseca 2009 |  |  |  |  |  | X |  |  |  |  |  |  |  |
| Schweikert 2006 |  |  |  | X | X |  |  |  |  |  |  | X |  |
| Sheeran 2016 |  | X |  |  |  |  |  |  |  |  |  |  |  |
| Siemonsma 2013 |  |  |  |  |  | X |  |  |  |  |  |  |  |
| Smeets 2006 |  |  |  | X | X |  | X |  | X |  |  | X |  |
| Smeets 2008 |  |  |  |  |  | X | X |  |  |  |  |  |  |
| Smeets 2009 |  |  |  |  |  |  | X |  |  |  |  |  |  |
| Soleymani 2021 |  |  |  |  |  | X |  |  |  |  |  |  |  |
| Spinhoven 2004 |  |  |  |  |  | X |  |  |  |  |  |  |  |
| Strong 1998 |  |  |  |  | X |  |  |  |  |  |  |  |  |
| Turner 1982 |  |  |  |  | X | X |  |  |  |  |  |  |  |
| Turner 1988 |  |  |  |  | X | X |  |  |  |  | X |  |  |
| Turner 1990 |  |  |  | X | X |  |  |  |  |  |  |  | X |
| Turner 1993 |  |  |  |  | X | X |  |  |  |  | X |  |  |
| Turner 2016 |  |  |  |  |  |  |  | X |  |  |  | X |  |
| Van der Hout 2003 |  |  |  |  | X |  |  |  |  |  |  |  |  |
| Van der Roer 2008 |  |  |  |  |  |  | X |  |  |  |  |  | X |
| Van Erp 2021 |  |  |  |  |  |  |  |  |  |  |  |  | X |
| Vibe Fersum 2013 |  | X |  |  |  |  |  |  |  |  |  |  | X |
| Vibe Fersum 2019 |  |  |  |  |  | X |  |  |  |  |  |  |  |
| Vlaeyen 2001 |  |  |  |  |  |  | X |  |  |  |  |  |  |
| Vlaeyen 2002 |  |  |  |  |  |  | X |  |  |  |  |  |  |
| Vong 2011 |  |  |  | X |  |  |  |  |  |  |  |  |  |
| Woods 2008 |  |  |  |  |  | X | X |  |  |  |  |  |  |
| Zgierska 2016 | X |  |  |  |  |  |  |  | X |  |  |  |  |
| **Total** | 5 | 5 | 1 | 10 | 21 | 32 | 12 | 3 | 13 | 4 | 5 | 22 | 18 |

**S4 Table. Citation Matrix of Primary Studies.**

Grey columns – Systematic reviews with 100% overlap of primary studies
