# Supplementary material for: The effect of cognitive behavioural therapy on pain and disability in chronic non-specific low back pain: An overview of systematic reviews
Source: PLoS One. 2025 Jun 17;20(6):e0325122. doi: 10.1371/journal.pone.0325122 (PMC12173195; doi:10.1371/journal.pone.0325122)
Supplement: S6 Table — (DOCX) [file pone.0325122.s006.docx]

| **Reviews** | **Quantitative Results** | **Narrative Results** | **Certainty of Evidence** |
| --- | --- | --- | --- |
| Bahnamiri, 2020 | No meta-analysis | Pain:  MBSR vs CBT: no significant difference  MBCT vs CBT: no significant difference  MBCT vs usual care: significant difference  Mindfulness meditation-CBT vs UC: significant difference | Not done |
| Devonshire, 2023 | Meta-analysis (2 studies)  Pain (NRS): Post intervention -1.02 (95% CI -14.75 to 12.7), I^2^ = 93.6%  12 months -0.55 (95% CI -12.62 to 11.52), I^2^ = 90.3%  Disability (ODI): Post intervention -6.95 (95% CI -12.62 to 11.52), I^2^ = 89.8%  12 months -4.62 (95% CI -70.31 to 61.07), I^2^ = 90.8% | Pain: no significant difference post intervention and at 12 months  Disability: no significant difference post intervention and at 12 months  General trend favours CFT in reducing pain intensity and disability compared to manual therapy/exercise | GRADE  Pain – Very low  Disability – Very low  Ranked down 2 from high risk of bias, 1 from imprecision, 1 from inconsistency, 1 from indirectness |
| Hajihasani, 2019 | No meta-analysis | Pain: 5 studies showed significant difference where CBT + PT is more effective than PT alone  Disability: 4 studies showed significant difference where CBT + PT is more effective than PT alone | Not done |
| Henschke, 2010  *Cochrane Review* | Meta-analysis (13 studies total)  Pain: CBT compared to WL – SMD -0.6 (95% CI -0.97 to -0.22), I^2^ = 42.49%  CBT + PT compared to PT short term – SMD -0.13 (95% CI -1.01 to 0.75), I^2^ = 73.02%  CBT vs exercise long term – MD 0.14 (95% CI -4.4 to 4.67), I^2^ = 0%  Disability: CBT vs WL SMD -0.36 (95% CI -0.87 to 0.13), I^2^ = 49.6%  CBT + PT compared to PT short term – MD -6.26 (95% CI -12.71 to 0.19), I^2^ = 0%  *note: not all results are included as there are too many | Pain:  CBT compared to WL: significant, favours CBT  CBT compared to behavioural therapy: not significant, general trend favours CBT compared to cognitive therapy and operant therapy; favours respondent therapy compared to CBT  CBT compared to group exercise: not significant  CBT + PT compared to PT: not significant  CBT + rehab compared to rehab: not significant  Disability:  CBT compared to WL: not significant  CBT compared to behavioural therapy: not significant, general trend favours CBT compared to cognitive therapy and operant therapy; favours respondent therapy compared to CBT  CBT compared to surgery: not significant, favours surgery  CBT + PT compared to PT: not significant | GRADE  Pain – ranges from very low to moderate  Disability – ranges from very low to low  Ranked down for limitations in study design, imprecision and inconsistency |
| Ho,  2022 | Network Meta-analysis (25 studies)  Post intervention results compared to PT:  Pain: CBT + PT – SMD 0.92 (95% CI 0.43 – 1.42)  CBT – SMD 0.23 (95% CI -0.47 – 0.94)  Disability: CBT + PT – SMD 1.01 (95% CI 0.58 – 1.44)  CBT – SMD 0.39 (95% CI -0.30 – 1.08) | Pain:  CBT + PT have moderate significant effect compared to PT at post intervention and at short term, no longer significant at mid and long term  CBT alone has small but non-significant effect compared to PT  Disability:  CBT + PT have significant effect compared to PT at post intervention and short term, no longer significant at mid to long term  CBT alone has small but non-significant effect compared to PT  Comparison to other interventions using SUCRA Mean rank:  Pain: CBT + PT rank 2nd post intervention, 1st long term; CBT ranks 5th post intervention, 3rd long term  Disability: CBT + PT rank 1st for post treatment and long term, CBT ranks 4th post treatment, 6th long term | CINeMA  Pain – moderate  Disability – moderate  Concerns in heterogeneity and within study bias |
| Lopez-de-Uralde-Villanueva, 2016 | Meta-analysis (8 studies)  Disability:  GA compared to control: Short term – SMD -0.3 (95% CI -0.55 to -0.05), Q value = 5.7  Long term – SMD -0.53 (95% CI -0.79 to -0.27), Q value = 1.6  GA compared to GEXP: short term – SMD 0.39 (95% CO 0.003 to 0.78), Q value = 1.1 | Pain:  Compared to other exercise: no significant difference  Compared to control: no significant difference  Compared to GEXP: no significant difference  Disability:  Compared to other exercise: no significant difference  Compared to control: significant difference in short and long term, favours GA  Compared to GEXP: significant difference in short term, favours GEXP | Van Peppen et al 2004  Disability: Limited evidence for reduction in disability between GA and control; Strong evidence for no difference between GA and GEXP  Pain: Strong evidence for no difference between GA and GEXP |
| Petrucci,  2021 | Meta-analysis (13 studies)  CBT compared to control:  Pain: SMD -0.73 (95% CI -1.2 to -0.26), I^2^ = 93%  Disability: SMD -0.88 (95% CI -1.5 to -0.26), I^2^ = 95%  CBT compared to MBSR:  Pain: MD -0.05 (95% CI -0.5 to 0.39) | Pain:  CBT shows significant improvement compared to control  CBT shows no significant difference compared to MBSR  Disability:  CBT shows significant improvement compared to control | Not done |
| Rihn,  2017 | Meta-analysis (4 studies)  Disability: TDR vs exercise+CBT MD -5.9 (95% CI -9.03 to -2.77)  Indirect comparison from multiple treatment analysis:  Pain: Exercise + CBT vs PT MD-15.81 (95% CI -23.72 to -7.42)  Disability: Exercise + CBT vs PT MD -6.8 (-12.77 to -1.49) | Pain: not significant for TDR or fusion  Disability: significant for TDR but not significant for fusion  General trend favours both surgery types  Likelihood of intervention superiority from multiple treatment analysis:  Exercise+CBT rank lower than TDR and fusion, ranks higher than PT from indirect comparison | GRADE  Pain: Moderate for TDR; Low for fusion  Disability: High for TDR; Low for fusion  Ranked down for imprecision and inconsistency |
| Yang,  2022 | Meta-analysis (16 studies)  Post intervention  Pain: SMD -0.32 (95% CI -0.57 to -0.06), I^2^ = 87%  Disability: SMD -0.44 (-0.71 to -0.17), I^2^ = 89%  Subgroup analysis:  Pain: CBT+control vs control (concurrent) SMD -0.67 (95% CI -1.21 to -0.13), I^2^ = 94%  Disability: Concurrent CBT SMD -0.81 (95% CI -1.35 to -0.27), I^2^ = 95%;  CBT vs UC/WL SMD -0.34 (95% CI -0.56 to -0.12), I^2^ = 37% | Pain: CBT shows significant improvement post intervention but no longer significant at 3 months and onwards  Disability: CBT shows significant effect post intervention but no longer significant at 3 months and onwards  Subgroup analysis:  Pain: significant effect for concurrent CBT only, but not significant when compared to UC/WL or active treatment  Disability: significant effect for concurrent CBT and compared to UC/WL, but not significant compared to active treatment | GRADE  Pain: Low  Disability Low  Ranked down for risk of bias, imprecision and inconsistency |
| Jurak, 2023 | Meta-analysis (18 studies)  Pain: MBR-BE vs MI – MD 17.17 (95% CI 11.34 to 22.99)  MBR-BE vs UC – MD 12.36 (95% CI 7.52 to 17.20)  Disability: MBR-BE vs MI – MD 0.88 (95% CI 0.46 to 1.30)  MBR-BE vs UC – MD 0.81 (95% 0.49 to 1.13) | Pain: significant effect against MI and UC, but not significant against exercise, MBR-work rehabilitation and MBR-education  Disability: significant effect against MI and UC, but not significant against exercise, MBR-work rehabilitation and MBR-education  Comparison to other interventions using P scores:  Pain: MBR-BE ranks 2nd  Disability: MBR-BE ranks 1st | Not done |

**S6 Table. Results of Included Systematic Reviews.**
