# Supplementary material for: The effect of cognitive behavioural therapy on pain and disability in chronic non-specific low back pain: An overview of systematic reviews
Source: PLoS One. 2025 Jun 17;20(6):e0325122. doi: 10.1371/journal.pone.0325122 (PMC12173195; doi:10.1371/journal.pone.0325122)
Supplement: S7 Table — (DOCX) [file pone.0325122.s007.docx]

| **Comparisons** | **Bahnamiri** | **Devonshire** | **Hajihasani** | **Henschke** | **Ho** | **Lopez-de-Uralde-Villanueva** | **Petrucci** | **Rihn** | **Yang** | **Jurak** |
| --- | --- | --- | --- | --- | --- | --- | --- | --- | --- | --- |
| CBT vs WL/UC |  |  |  | X |  |  |  |  | X |  |
| CBT vs AT | X |  |  | X | X |  | X |  | X |  |
| CBT + exercise vs Surgery |  |  |  | X |  |  |  | X |  |  |
| CBT + AT vs same AT |  |  | X | X | X |  |  |  | X |  |
| Multidisciplinary programs vs WL/UC |  |  |  |  |  |  |  |  |  | X |
| Multidisciplinary programs vs AT |  |  |  | X |  |  |  |  |  | X |
| GA vs WL/UC |  |  |  |  |  | X |  |  |  |  |
| GA vs AT |  |  |  |  |  | X |  |  |  |  |
| CFT vs exercise/ manual therapy |  | X |  |  |  |  |  |  |  |  |
| Mindfulness + CBT vs UC | X |  |  |  |  |  |  |  |  |  |
| Mindfulness + CBT vs CBT | X |  |  |  |  |  |  |  |  |  |

**S7 Table. Systematic reviews involved in each comparison.**
