# Supplementary material for: The effect of cognitive behavioural therapy on pain and disability in chronic non-specific low back pain: An overview of systematic reviews
Source: PLoS One. 2025 Jun 17;20(6):e0325122. doi: 10.1371/journal.pone.0325122 (PMC12173195; doi:10.1371/journal.pone.0325122)
Supplement: Attachment — Submitted filename: PONE-D-24-51702_reviewer.pdf [file pone.0325122.s008.pdf]

PLOS ONE

The effect of cognitive behavioural therapy on pain and disability in chronic non-specific low back pain: an overview of systematic reviews

--Manuscript Draft--

**Manuscript Number:**	PONE-D-24-51702
**Article Type:**	Research Article
**Full Title:**	The effect of cognitive behavioural therapy on pain and disability in chronic non-specific low back pain: an overview of systematic reviews
**Short Title:**	Cognitive behavioural therapy in low back pain
**Corresponding Author:**	Ana-Catarina Pinho-Gomes<br>UCL: University College London<br>London, UNITED KINGDOM OF GREAT BRITAIN AND NORTHERN IRELAND
**Keywords:**	chronic non-specific low back pain; cognitive behavioural therapy
**Abstract:**	Background: Low back pain (LBP) is experienced by most people at least once in their lifetime and is the leading cause of disability worldwide. Cognitive behavioural therapy (CBT) is a non-invasive method used to treat chronic non-specific low back pain (CNSLBP) and contains less adverse effects than analgesic medications.<br>Aims: To determine the efficacy of interventions incorporating CBT on reducing pain intensity and physical disability in CNSLBP.<br>Methods: An overview of reviews without meta-analysis was conducted. 4 electronic databases (MEDLINE, Embase, Cochrane Library and PsycINFO) were searched. Adults aged 18 or above with CNSLBP were included. AMSTAR 2 was used to assess risk of bias of included systematic reviews and data synthesis was done via vote counting methods.<br>Results: 10 systematic reviews were included. Results showed that CBT is more effective in reducing pain and disability than waiting list/usual care (WL/UC). CBT in conjunction with other active treatments (AT) are also more effective than some standalone active treatments such as physiotherapy and exercise, but less effective than surgery.<br>Conclusion: CBT has some effect in reducing pain and disability, however, may not be more effective than other treatments. The quality of evidence was low for most outcomes as there was high risk of bias and high heterogeneity between studies. Future research could evaluate specific therapies such as acceptance and commitment therapy (ACT) and mindfulness-based cognitive therapy (MBCT), as these treatments lacked primary studies. There is no recognized standard for conducting CBT, and further research could determine the most effective duration, intensity of CBT and any differences in delivery of CBT (e.g. online, group-based, etc.).<br>Funding/Competing Interests: None.
**Order of Authors:**	Tiffany Leung
	Mei Ken Low
	Pei Chi Yao
	Ana-Catarina Pinho-Gomes
**Additional Information:**	
**Question**	**Response**
**Financial Disclosure**<br><br>Enter a financial disclosure statement that describes the sources of funding for the work included in this submission. Review the submission guidelines for detailed requirements. View published research articles from *PLOS ONE* for specific	The author(s) received no specific funding for this work.

Powered by Editorial Manager® and ProduXion Manager® from Aries Systems Corporation

examples.<br><br>This statement is required for submission and **will appear in the published article** if the submission is accepted. Please make sure it is accurate.<br><br>**Funded studies**<br>Enter a statement with the following details:<br>• Initials of the authors who received each award<br>• Grant numbers awarded to each author<br>• The full name of each funder<br>• URL of each funder website<br>• Did the sponsors or funders play any role in the study design, data collection and analysis, decision to publish, or preparation of the manuscript?<br><br>Did you receive funding for this work?	
**Competing Interests**<br><br>Use the instructions below to enter a competing interest statement for this submission. On behalf of all authors, disclose any competing interests that could be perceived to bias this work—acknowledging all financial support and any other relevant financial or non-financial competing interests.<br><br>This statement is required for submission and **will appear in the published article** if the submission is accepted. Please make sure it is accurate and that any funding sources listed in your Funding Information later in the submission form are also declared in your Financial Disclosure statement.<br><br>View published research articles from *PLOS ONE* for specific examples.	The authors have declared that no competing interests exist.

**NO authors have competing interests**<br><br>Enter: *The authors have declared that no competing interests exist.*<br><br>**Authors with competing interests**<br><br>Enter competing interest details beginning with this statement:<br><br>*I have read the journal's policy and the authors of this manuscript have the following competing interests: [insert competing interests here]*<br><br>* typeset	
**Ethics Statement**<br><br>Enter an ethics statement for this submission. This statement is required if the study involved:<br><br>• Human participants<br>• Human specimens or tissue<br>• Vertebrate animals or cephalopods<br>• Vertebrate embryos or tissues<br>• Field research<br><br>Write "N/A" if the submission does not require an ethics statement.<br><br>General guidance is provided below. Consult the submission guidelines for detailed instructions. **Make sure that all information entered here is included in the Methods section of the manuscript.**	N/A

**Format for specific study types**<br><br>**Human Subject Research (involving human participants and/or tissue)**<br>• Give the name of the institutional review board or ethics committee that approved the study<br>• Include the approval number and/or a statement indicating approval of this research<br>• Indicate the form of consent obtained (written/oral) or the reason that consent was not obtained (e.g. the data were analyzed anonymously)<br><br>**Animal Research (involving vertebrate animals, embryos or tissues)**<br>• Provide the name of the Institutional Animal Care and Use Committee (IACUC) or other relevant ethics board that reviewed the study protocol, and indicate whether they approved this research or granted a formal waiver of ethical approval<br>• Include an approval number if one was obtained<br>• If the study involved *non-human primates*, add *additional details* about animal welfare and steps taken to ameliorate suffering<br>• If anesthesia, euthanasia, or any kind of animal sacrifice is part of the study, include briefly which substances and/or methods were applied<br><br>**Field Research**<br><br>Include the following details if this study involves the collection of plant, animal, or other materials from a natural setting:<br>• Field permit number<br>• Name of the institution or relevant body that granted permission	
**Data Availability**<br><br>Authors are required to make all data underlying the findings described fully available, without restriction, and from the time of publication. PLOS allows rare exceptions to address legal and ethical concerns. See the PLOS Data Policy and FAQ for detailed information.	Yes - all data are fully available without restriction

A Data Availability Statement describing where the data can be found is required at submission. Your answers to this question constitute the Data Availability Statement and **will be published in the article**, if accepted.<br><br>**Important:** Stating 'data available on request from the author' is not sufficient. If your data are only available upon request, select 'No' for the first question and explain your exceptional situation in the text box.<br><br>Do the authors confirm that all data underlying the findings described in their manuscript are fully available without restriction?	
**Describe where the data may be found in full sentences. If you are copying our sample text, replace any instances of XXX with the appropriate details.**<br><br>• If the data are **held or will be held in a public repository**, include URLs, accession numbers or DOIs. If this information will only be available after acceptance, indicate this by ticking the box below. For example: *All XXX files are available from the XXX database (accession number(s) XXX, XXX.).*<br>• If the data are all contained **within the manuscript and/or Supporting Information files**, enter the following: *All relevant data are within the manuscript and its Supporting Information files.*<br>• If neither of these applies but you are able to provide **details of access elsewhere**, with or without limitations, please do so. For example:<br><br>*Data cannot be shared publicly because of [XXX]. Data are available from the XXX Institutional Data Access / Ethics Committee (contact via XXX) for researchers who meet the criteria for access to confidential data.*<br><br>*The data underlying the results presented in the study are available from (include the name of the third party*	All relevant data are within the manuscript and its Supporting Information files.

and contact information or URL).<br>• This text is appropriate if the data are owned by a third party and authors do not have permission to share the data.<br><br>* typeset	
Additional data availability information:	

Manuscript Click here to access/download;Manuscript;Publication final.docx

The effect of cognitive behavioural therapy on pain and disability in chronic non-specific low back pain: an overview of systematic reviews

Tiffany LEUNG[1], Mei Ken LOW[2], Pei Chi YAO[1], Ana-Catarina PINHO-GOMES[3*]

[1]Institute of Epidemiology and Health Care, University College London, London, United Kingdom

[2]Institute of Ophthalmology, University College London, London, United Kingdom

[3]Institute for Global Health, University College London, London, United Kingdom

[*]Corresponding author

Email: a.pinho-gomes@ucl.ac.uk

Abstract

Background: Low back pain (LBP) is experienced by most people at least once in their lifetime and is the leading cause of disability worldwide. Cognitive behavioural therapy (CBT) is a non-invasive method used to treat chronic non-specific low back pain (CNSLBP) and contains less adverse effects than analgesic medications.

Aims: To determine the efficacy of interventions incorporating CBT on reducing pain intensity and physical disability in CNSLBP.

Methods: An overview of reviews without meta-analysis was conducted. 4 electronic databases (MEDLINE, Embase, Cochrane Library and PsycINFO) were searched. Adults aged 18 or above with CNSLBP were included. AMSTAR 2 was used to assess risk of bias of included systematic reviews and data synthesis was done via vote counting methods.

Results: 10 systematic reviews were included. Results showed that CBT is more effective in reducing pain and disability than waiting list/usual care (WL/UC). CBT in conjunction with other active treatments (AT) are also more effective than some standalone active treatments such as physiotherapy and exercise, but less effective than surgery.

Conclusion: CBT has some effect in reducing pain and disability, however, may not be more effective than other treatments. The quality of evidence was low for most outcomes as there was high risk of bias and high heterogeneity between studies. Future research could evaluate specific therapies such as acceptance and commitment therapy (ACT) and mindfulness-based cognitive therapy (MBCT), as these treatments lacked primary studies. There is no recognized standard for

conducting CBT, and further research could determine the most effective duration, intensity of CBT and any differences in delivery of CBT (e.g. online, group-based, etc.).

Funding/Competing Interests: None.

Introduction

Low back pain (LBP) is a common medical condition that describes pain arising from the 12th ribs down to the hip and buttock area (1, 2). The predominant complaints of having LBP are pain and disability. LBP is the biggest cause of disability in the world, leading to a loss of workforce productivity (3). People with LBP often have a lower quality of life and suffer from poor mental health due to inability to engage in regular work and social activities (4).

In 85-90% of people with LBP (5), this pain is not attributable to underlying medical conditions or pathology – this is termed as non-specific (1). It is speculated to be musculoskeletal in nature, arising from the overuse or strain of back muscle groups (5).

Most people experience LBP at least once in their lives (3). It is estimated that in the UK, the 1-month period prevalence of LBP is 28.5%. It peaks between ages of 41-50 at 29.8%, though it affects people of all age groups. This falls to 25% at 80 years of age, however the severity of LBP increases with age (6). According to the UK Office of National Statistics, there were more than 250 000 people of working age who were economically inactive due to back or neck problems in 2022 (7). This results in both direct healthcare and indirect absent workforce costs to the economy, and is estimated to cost more than £10 billion (8).

As per National Institute for Health and Care Excellence (NICE) guidelines, current management methods for LBP consist of pharmacological, invasive and non-invasive treatments (9). Pharmacological treatment focuses on reducing pain intensity, but have numerous adverse effects and reduced efficacy with repeated use (10). Invasive treatments include back injections and surgery – these treatments are normally reserved for people with moderate to severe LBP

and come with procedural risks, such as infection, bleeding and damage to local anatomical structures (11).

Non-invasive treatment methods include self-management, exercise and psychological therapies (9). There is little detail in NICE guidelines regarding psychological treatments; currently they are only recommended if in combination with physical activity and incorporate a cognitive behavioural approach.

Cognitive behavioural therapy (CBT) is the most well-known psychological therapy and is part of the “second wave” of behavioural therapy for managing psychiatric disorders. Whilst CBT does not remove the source of pain, it encourages a problem-solving attitude by helping people develop strategies to alter their negative perception of pain and increase physical function (12, 13). By allowing people to adapt and cope better with discomfort, chronic pain patients develop a higher tolerance for pain and reduction in behaviour of limiting physical activity. People have actually reported feeling less pain and improved functionality (12, 14).

More modern forms of CBT have been developed recently, bringing about the “third wave” of behavioural therapy. The third “wave” integrates other concepts such as metacognition, mindfulness, acceptance, personal values and goals into CBT (4, 15). Examples of the third “wave” include mindfulness-based cognitive therapy (MBCT) and acceptance and commitment therapy (ACT) (15). With the advancement of psychological therapies, it is important to re-examine how treatments incorporating cognitive behavioural approaches affect LBP and how it compares to other management methods.

The existence of CBT spans a few decades and a preliminary search revealed that there are numerous systematic reviews and literature reviews published on assessing the efficacy of CBT in treating LBP (16, 17). For this reason, an overview of reviews was considered to be appropriate to synthesize evidence from multiple systematic reviews on this topic. An overview of reviews is similar to a systematic review, with the difference in the unit of interest being systematic reviews instead of RCTs, non-randomised studies or observational studies (18).

Methods

This overview was written following the guidance of Cochrane Handbook of Systematic Reviews (19) and Synthesis without meta-analysis (SWiM) guidelines (20). A protocol was submitted to PROSPERO (CRD42024527690) on 26th March 2024, and the PRISMA checklist is shown in S1 and S2 Checklist (21).

Search Strategy

A search of eligible systematic reviews was conducted in 4 electronic databases – MEDLINE, Embase, Cochrane Library and PsycINFO. Clinical trial registers were not applicable to this overview as the units of interest were systematic reviews instead of individual trials. The search strategy was comprised of Medical Subject Headings (MeSH), keywords and phrases that were similar to "low back pain", "CBT", "systematic review". The full search strategy is in S3 Supporting Information.

Eligibility Criteria

Population

The study population was adults aged 18 years or older, with chronic non-specific low back pain (CNSLBP) lasting at least 3 months (22).

Interventions

Interventions were any therapies that incorporated a cognitive behavioural approach. This included standalone CBT and any treatment that contains a cognitive behavioural element.

Examples of such treatments were multidisciplinary programs, cognitive functional therapy (CFT), graded activity (GA), MBCT and ACT.

Comparisons

All comparison groups were eligible for inclusion and were divided into passive control or active treatment (AT). No treatment, sham treatment, waiting list (WL) or usual care (UC) were included in passive control. All others were considered as AT.

Outcomes

The primary outcomes of interest were pain intensity and disability. At least one of the two must be a primary outcome in the systematic review to be eligible for inclusion. In some reviews, disability was referred to as physical function.

Exclusion criteria

Known inflammatory, infectious, neurological or neoplastic causes of LBP and spinal injuries were excluded. Pain duration of less than 3 months and population including individuals younger than 18 years old were not eligible. Systematic reviews that analysed mixed psychological interventions or mixed chronic pain together were excluded.

Study Selection

The search and study screening were conducted by TL and ML, from inception of each database up to 20th April 2024. All disagreements were screened by a third reviewer PY. Results of the search were deduplicated using EndNote 20 (23), following Jane Falconer's approach from London School of Hygiene and Tropical Medicine (24). After deduplication, results were exported

to Rayyan for title and abstract screening (25). Texts eligible for full text screening were exported back to EndNote 20 (23).

The database search was not restricted by language; however, texts were excluded at full text screening if no English translation was found. For protocols that met the inclusion criteria, a search for a complete published review was conducted.

Multiple systematic reviews that used the same clinical trials would affect the results of an overview of reviews. A decision tool from Pollock et al. (26) was used to decide which systematic review to include. All primary studies were listed and entered in a citation matrix to identify any overlapping primary studies. The corrected covered area (CCA) was calculated, as per Pieper et al. (27). CCA ≤5% was considered slight overlap and CCA ≥15% was considered high overlap. Systematic reviews that had 100% overlap of primary studies with other systematic reviews were excluded, following the guidance from Hennessy et al. (28)

Data Extraction

A custom Excel spreadsheet was created for data extraction, performed by TL and ML. All data for search strategy (search database, search date), publication details (number of studies included, publication year, funding and conflict of interest), participants (including age, gender), interventions and comparisons (duration, intensity, setting), outcomes, results (qualitative and quantitative), risk of bias tools, certainty of evidence were collected.

Assessment of Methodological Quality

The Risk of Bias assessment was conducted by TL and PY, using the AMSTAR 2 instrument from Shea et al 2017 (29). The rating of the overall confidence of results depended on the number of critical and non-critical domains satisfied.

Data Synthesis

Interventions were arranged in the following groups: standalone CBT, CBT in combination with AT, multidisciplinary programs involving CBT and specific third "wave" therapies that incorporate cognitive behavioural elements such as CFT and MBCT.

A qualitative synthesis was conducted using a vote counting method based on direction of effect, following guidance from Cochrane Handbook (19) and Boon et al (30). A direction was reported if there was ≥70% of studies supporting a treatment. If there was <70% of studies, this was considered indeterminate direction.

Other synthesis methods was not appropriate in this overview (19). The heterogeneity in outcome measurements across different systematic reviews have made it difficult for quantitative synthesis. Moreover, not all systematic reviews provided an estimate of effect, precise p-value, or conducted a meta-analysis.

Results

This overview of reviews ultimately included 10 systematic reviews, as seen in Fig 1 showing the PRISMA flow diagram (21).

Fig 1. PRISMA flow diagram

The search was conducted on 20th April 2024. Of the 94 full texts screened, 12 protocols were included, and 4 protocols had full publications included within the 94 full texts. Out of 82 full publications, 13 were eligible. The primary studies of the 13 systematic reviews were all RCTs. Non-Cochrane reviews were included in this overview as there was only one Cochrane review eligible for inclusion.

A citation matrix was done to identify overlapping primary studies, see S4 Table. CCA was 6.5%, indicating there is low to moderate degree of overlap. 3 of the 13 systematic reviews were found to have 100% overlap of primary studies with other systematic reviews, were further excluded from analysis (31-33).

Table 1 shows the characteristics of each individual systematic review.

Table 1. Characteristics of included reviews

Reviews	Participants	Intervention	Comparison	Primary Outcomes	Follow-up time points
Bahnamiri, 2020 (34)	543 (5 studies)<br>Age: average 48.09 to 54.26<br>Gender: not available	Mindfulness-based interventions, such as MBSR, MBCT<br>90-120 min session for 8 weeks, with homework	UC;<br>CBT 90-120 min session for 8 weeks, with homework	Pain (BPI/NRS/MPQ)	Post intervention, 3, 6 and 12 months
Devonshire, 2023 (35)	507 (5 studies)<br>Intervention: 262<br>Control: 245<br>Age: mean 44.6 (range 40.4-50.6)<br>Gender: 38% male	CFT – integrated behavioural approach led by physiotherapists to manage pain<br>30 min to 1 hour session, over 8-13.7 weeks<br>Delivered by physiotherapists	Physical therapy;<br>Exercise programs	Pain (NRS/VAS)<br>Disability (ODI)	Post intervention, 6 and 12 months
Hajihasani, 2019 (36)	965 (10 studies)<br>Intervention: 484<br>Control: 481<br>Age: range 18-65<br>Gender: not available	CBT + AT:<br>Exercise, physiotherapy or education<br>2-3 session per week over 3-12 weeks	Same AT as intervention group:<br>Exercise, physiotherapy or education	Pain (VAS/NRS/MPQ)<br>Disability (RMDQ/LBOS/LBP rating)<br>Quality of life, depression, functional capacity	Not available
Henschke, 2010 (37)<br>*Cochrane Review*	2674 (21 studies)<br>Intervention: 1442<br>Control: 1035<br>Age: range 18-65<br>Gender: not available	CBT ± physiotherapy/ rehabilitation program over 3-12 weeks<br>Delivered at primary and secondary health centres	WL control; Exercise;<br>Other behavioural therapy;<br>Physiotherapy; Rehabilitation program	Pain (VAS/NRS/MPQ)<br>Disability (RMDQ/ODI/ QBPDS/ADL/sickness impact profile)	Short (post intervention)<br>Intermediate (6 months)<br>Long term (12 months)
Ho, 2022 (38)	3851 (33 studies)<br>Intervention: 1792<br>Control: 2059<br>Age: mean 37.67 to 54<br>Gender: 8.33-65.68% male	CBT over 4-10 weeks;<br>CBT + physiotherapy over 3-52 weeks<br>Delivered in outpatient settings	Physiotherapy;<br>Other comparisons were not examined in network meta-analysis, including education, usual care, etc.	Pain (NRS/VAS/Box scale)<br>Disability (ODI/RMDQ/PDI)	Post intervention (0-2 months)<br>Short (2-6 months)<br>Mid (6-12 months),<br>Long term (≥12 months)
Lopez-de-Uralde-Villanueva, 2016 (39)	1486 (12 studies)<br>Age: average 35-49<br>Gender: 25-58% male	GA – exercise using cognitive behavioural approach to improve activity tolerance by setting feasible goals at a rate manageable by participants	Exercise; Physiotherapy; WL; crossover study with GEXP – similar to GA but exposes individuals to fearful situations instead of goal setting	Pain (VAS/NRS/MPQ)<br>Disability (RMDQ/QBPD/ PDI)<br>Pain catastrophising, quality of life	Short (<3 months)<br>Intermediate (3-12 months)<br>Long term (>12 months)

Petrucci, 2021 (40)	1684 (13 studies)<br>Intervention: 883<br>Control: 801<br>Age: mean 39.61 – 64.3<br>Gender: 20-92% male	CBT ± exercise/medical treatment/education	UC; WL;<br>Same active treatment as intervention group	Pain (VAS/NRS/BPI)<br>Disability (RMDQ/ODI/ PROMIS)<br>Depression, anxiety	Mean 7.8 months, range from 3 weeks to 15 months
Rihn, 2017 (41)	646 (4 studies)<br>Intervention: 328<br>Control: 318<br>In moderate to severe pain or disability (pain ≥ 5, ODI ≥ 30), degenerative changes in intervertebral discs at lumbar levels<br>Age: mean 39.4-44.1<br>Gender: 39-52% male	Spinal fusion;<br>Prodisc-L total disk replacement	Exercise + CBT for 60-110 hours over 3-5 weeks	Pain (VAS)<br>Disability (ODI),<br>Complication risks, additional surgeries, cost-effectiveness	12 and 24 months
Yang, 2022 (42)	3003 (22 studies)<br>Age: not available<br>Gender: 16.8-93.4% male	CBT; ACT;<br>CBT + AT:<br>UC, physiotherapy, exercise, rehabilitation program<br>Mean duration 10 weeks, 2-18 sessions lasting 30 to 120 mins each<br>Majority is face to face, internet based, but also includes telephone, text and audiotape	UC; WL; Biofeedback;<br>Same AT as intervention group: physiotherapy, exercise, rehabilitation program, brief intervention	Pain (NRS/VAS)<br>Disability (RMDQ/ODI/ADL)	Post intervention, 3, 6 and 12 months
Jurak, 2023 (43)	1662 (18 studies)<br>Age: average 44.61 (21.4 to 73.63)<br>Gender: Male 31.18%	Multidisciplinary biopsychosocial rehabilitation with behavioural, education or work-related component<br>Mix of inpatient and outpatient	Minimal intervention; UC; exercise	Pain (VAS/MPQ/NRS)<br>Disability (RMDQ/QBPD/ODI/PDI/ Hanover functional abilitys)	Mostly 8 weeks, range from 2 to 12 weeks

ACT, acceptance and commitment therapy; ADL, activity of daily living; AT, active treatment; BPI, brief pain inventory; CFT, cognitive functional therapy; LBOS, low back outcome scale; MBCT, mindfulness-based cognitive therapy; MBSR, mindfulness-based stress reduction; MPQ, McGill pain questionnaire; NRS, numeric rating scale; ODI, Oswestry disability index; PDI, pain disability index; PROMIS, patient-reported outcomes measurement information system; QBPD, Quebec Back Pain Disability; RMDQ, Roland Morris Disability Questionnaire; UC, usual care; VAS, visual analogue scale; WL, waiting list

Methodological Quality

Table 2 summarises the methodological quality of each systematic review. 7 systematic reviews used a risk of bias tool that was established by Cochrane (44, 45). More than half of the included primary RCTs are high risk or had some concern, where the most common domains of weakness were measurement of outcomes and lack of blinding in participants. Most RCTs had low risk in selection bias, randomization process, allocation concealment and selective reporting.

Funding and competing interests are declared in all systematic reviews. 2 systematic reviews had conducted their database search more than 2 years prior to publication.

Table 2. Methodological Quality within individual Systematic Reviews

Reviews	Number of studies	Last searched	Funding and Competing Interests	Risk of Bias Tool	Risk of Bias Assessment
Bahnamiri, 2020 (34)	5	2014 up to June 2020	No funding, no conflicts of interest	Cochrane Risk of Bias tool (ROB 2)	2 studies – low risk<br>3 studies – some concerns at randomization process, deviations from intended intervention and measurement of outcome
Devonshire, 2023 (35)	5	Up to March 2022	No financial involvement with any organisations, no competing interests	Cochrane Risk of Bias 2 Tool	All studies – high risk<br>Measurement of outcome and selection of reported result
Hajihasani, 2019 (36)	10	Up to January 2018	Support from Clinical Research Development Center of Rofeideh Rehabilitation Hospital and Neuromuscular Rehabilitation Research Center of Semnan University of Medical Sciences; authors disclosure: nothing to disclose	Hailey et al 2004 – Study assessment system	7 studies – high quality<br>3 studies – good quality<br>Areas of bias include inadequate description of randomization methods and small sample size
Henschke, 2010 (37) *Cochrane Review*	21	Up to February 2009	Multiple sources of support stated; 2 authors of this Cochrane review were co-authors of multiple included primary studies	Cochrane Back Review Group	10 studies – low risk<br>11 studies – high risk<br>Majority of bias from blinding of participants and outcome assessors, inadequate randomization procedure and allocation concealment, inadequate compliance
Ho, 2022 (38)	33	Up to 31 January 2021	No grant or financial relationship with any organisations, no competing interests	Cochrane Risk of Bias 2 tool	3 studies – high risk<br>Majority of studies – some concerns in measurement of outcome and selection of reported results
Lopez-de-Uralde-Villanueva, 2016 (39)	12	Up to 10 December 2013	No financial support, no conflicts of interest	PEDro scale	8 studies – good methodology<br>2 studies – moderate methodology<br>2 studies – poor methodology<br>Areas of bias include lack of binding of participants, care provider and assessor
Petrucci, 2021 (40)	13	Last 25 years	Research grant from Italian Workers' Compensation Authority (INAIL), no conflicts of interest	Cochrane Risk of Bias tool	9 studies – unclear<br>2 studies – high risk<br>2 studies – low risk<br>All had high risk for blinding of participants

Rihn, 2017 (41)	4	1990 up to January 2014	Grant from Association for Collaborative Spine Research, authors declared any financial payments or stock ownership interest	Journal of Bone & Joint Surgery criteria	All studies – moderately low risk<br>Areas of bias in blind assessment
Yang, 2022 (42)	22	1980 up to 20 November 2021	Research grant from National Natural Science Foundation of China, no conflicts of interest	Cochrane Back and Neck Review group	High risk of performance and detected bias
Jurak, 2023 (43)	18	Up to March 2022	1 author employed by "PhysioPlus" company, no financial support	Cochrane Risk of Bias 2 tool	8 studies – high risk<br>5 studies – some concerns<br>5 studies – low risk<br>Areas of bias include deviation from intended intervention and measurement of outcome, few had bias in selection of reported results

Results of Individual Systematic Reviews

The results of the individual systematic reviews are summarized in table 3. The full results can be found in S5 Table.

Table 3. Effect direction plot for results of individual reviews

Reviews	Intervention/Comparison	Primary studies	Pain Intensity	Primary studies	Disability
Bahnamiri, 2020 (34)	CBT vs MBSR	1	◀▶	-	-
	MBCT vs CBT	1	◀▶	-	-
	Mindfulness + CBT vs Usual care	3	▲	-	-
Devonshire, 2023 (35)	CFT vs exercise/manual therapy	5	△	4	△
Hajihasani, 2019 (36)	CBT + physiotherapy vs physiotherapy	5	▲	4	▲
Henschke, 2010 (37) *Cochrane Review*	CBT vs WL	5	▲	4	△
	CBT vs cognitive therapy	2	△	2	△
	CBT vs operant therapy	3	△	2	△
	CBT vs respondent therapy	3	▽	3	▽
	CBT vs group exercise	2	◀▶	-	-
	CBT + exercise vs surgery	-	-	2	▽
	CBT + Physiotherapy vs Physiotherapy	2	△	2	△
	CBT + rehab vs rehab	2	△	-	-
Ho, 2022 (38)	CBT vs Phystiotherapy	11	△	8	△
	CBT + Physiotherapy vs Physiotherapy	11	▲	11	▲
Lopez-de-Uralde-Villanueva, 2016 (39)	GA vs other exercise	4	△	6	△
	GA vs Usual Care/Waiting List (control)	4	△	4	▲
	GA vs GEXP	2	▽	2	▼
Petrucci, 2021 (40)	CBT vs control	11	▲	9	▲
	CBT vs MBSR	2	△	-	-
Rihn, 2017 (41)	CBT + exercise vs TDR	1	▽	1	▼
	CBT + exercise vs fusion	3	▽	3	▽
Yang, 2022 (42)	CBT vs Usual Care/Waiting List	5	△	6	△
	CBT vs active treatment	5	△	4	△
	Concurrent CBT	8	▲	9	▲
Jurak, 2023 (43)	MBR-BE vs MI	3	▲	3	▲
	MBR-BE vs Usual care	12	▲	12	▲
	MBR-BE vs Exercise	5	△	5	△

GEXP, graded exposure therapy; MBR-BE, multidisciplinary biopsychosocial rehabilitation – behavioural; MBSR, mindfulness-based stress reduction; MI, minimal intervention; TDR, total disc replacement; ▲, significant difference favouring intervention with CBT; △, non-significant difference favouring intervention with CBT; ▽, non-significant difference favouring comparison without CBT; ▼, significant difference favouring comparison without CBT; ◀▶, no change/mixed findings

Most of the results showed that interventions with CBT were favoured over comparison groups, however they lack statistical significance. Few treatment methods such as respondent therapy, surgery and GEXP were more effective in reducing pain intensity and disability compared to interventions with CBT.

A RCT by Rose et al. (46) was mentioned by two systematic reviews, however it was not included in Table 3 as the aim was to compare the efficacy of group vs individual treatment, and high vs low intensity treatment. There was no significant difference in either group and no direction of effect was given.

Three systematic reviews conducted multiple treatment comparisons. Ho et al. (38) conducted a network meta-analysis using the SUCRA mean rank to compare all psychological interventions in the management of CNSLBP. CBT with physiotherapy ranked first and is the most effective in reducing pain intensity in the long term, whereas standalone CBT ranked third in long term. For reducing disability, CBT with physiotherapy ranked first immediately after treatment and long term, and CBT ranked sixth in long term.

Rihn et al. (41) conducted a likelihood of intervention superiority from multiple treatment analysis. Direct comparison between surgery vs CBT with exercise showed both TDR and spinal fusion were more effective in reducing pain and disability in CNSLBP. Indirect comparison between physiotherapy vs CBT with exercise demonstrated that standalone physiotherapy was less effective than CBT with exercise in reducing pain and disability.

Jurak et al. (43) conducted a network meta-analysis using P-scores, where MBR-BE ranked first in reducing disability. MBR-BE ranked second in reducing pain.

Certainty of evidence

GRADE was extracted from individual systematic reviews, seen in S5 Table. Not all systematic reviews assessed certainty of evidence. 6 systematic reviews evaluated certainty of evidence, 4 of which used the GRADE tool (47). Most reviews showed low to moderate certainty, where outcomes were ranked down in the inconsistency and imprecision domains. These were largely due to performance bias, large heterogeneity between RCTs and small sample sizes.

Results of Data Synthesis

Data synthesis was conducted by vote counting of systematic reviews based on direction of effect. Table 4 shows the number of systematic reviews in each direction and overall direction for each comparison. The details of each comparison are shown in S6 Table.

Table 4. Qualitative Synthesis of Results

Interventions	Outcome	Favours intervention	Favours comparison	Indeterminate direction	Overall Direction
CBT vs WL/UC	Pain	2	0	0	▲
	Disability	2	0	0	▲
CBT vs AT	Pain	2	0	3	◄►
	Disability	3	0	1	▲
CBT + exercise vs surgery	Pain	0	1	0	▼
	Disability	0	2	0	▼
CBT + AT vs same AT	Pain	4	0	0	▲
	Disability	4	0	0	▲
Multidisciplinary program vs WL/UC	Pain	1	0	0	▲
	Disability	-	-	-	-
Multidisciplinary program vs AT	Pain	2	0	0	▲
	Disability	-	-	-	-
GA vs WL/UC	Pain	1	0	0	▲
	Disability	1	0	0	▲
GA vs AT	Pain	0	0	1	◄►
	Disability	0	0	1	◄►
CFT vs exercise/manual therapy	Pain	1	0	0	▲
	Disability	1	0	0	▲
Mindfulness + CBT vs UC	Pain	1	0	0	▲
	Disability	-	-	-	-
Mindfulness + CBT vs CBT	Pain	0	0	1	◄►
	Disability	-	-	-	-

AT, active treatment; CFT, cognitive functional therapy; GA, graded activity; WL/UC, waiting list/usual care; ▲, Studies overall favour intervention with CBT; ▼, Studies overall favour comparison without CBT; ◄►, no change/mixed findings

Table 4 shows that interventions with CBT are more effective than WL/UC, and the addition of CBT to AT is more effective than AT by itself. However, the direction of effect for interventions with CBT compared to AT is more varied. Direction for GA vs AT, and MBI with CBT vs CBT is indeterminate. Surgery is more effective in reducing pain intensity and disability than CBT with exercise.

There is a wide variety of AT, making results more difficult to interpret as it does not show which treatments are involved in this comparison. For the disability outcome in CBT + exercise vs surgery, the 2 systematic reviews use the same primary studies, which is a potential source of

bias. 3 out of 4 systematic reviews in CBT + AT vs same AT utilized physiotherapy as existing treatment and is another potential source of bias.

Causes of Heterogeneity

There is heterogeneity in population demographics, intensity and duration for each intervention and comparison. Furthermore, each primary study has different follow-up times and utilize different measurement scales for pain and disability. These are potential causes for heterogeneity in results.

Risk of Bias Assessment of Systematic Reviews

The AMSTAR 2 tool (29) is used to assess the methodological quality of included systematic reviews. Table 5 shows the results of AMSTAR 2, where yellow boxes indicate a critical domain. 7 reviews had low or critically low confidence. The most commonly missed critical domains were items 2 and 7, which would automatically result in critically low confidence. Multiple systematic reviews did not mention whether there were protocols registered or did not provide a list of excluded studies with justifications for exclusion.

Table 5. AMSTAR 2 quality assessment

**AMSTAR 2 Questions**	Bahnamiri	Devonshire	Hajihasani	Henschke	Ho	Lopez-de-Uralde-Villanueva	Petrucci	Rihn	Yang	Jurak
1. Did the research questions and inclusion criteria for the review include the components of PICO?	Yes	Yes	Yes	Yes	Yes	Yes	Yes	Yes	Yes	Yes
2. Did the report of the review contain an explicit statement that the review methods were established prior to the conduct of the review and did the report justify any significant deviations from the protocol?	No	Yes	No	Yes	Yes	No	Yes	No	Yes	Yes
3. Did the review authors explain their selection of the study designs for inclusion in the review?	No	No	No	No	No	No	No	Yes	No	No
4. Did the review authors use a comprehensive literature search strategy?	Yes	Yes	Yes	Yes	Yes	Yes	Yes	Yes	Yes	Yes
5. Did the review authors perform study selection in duplicate?	Yes	Yes	Yes	Yes	Yes	Yes	Yes	Yes	Yes	Yes
6. Did the review authors perform data extraction in duplicate?	Yes	Yes	No	Yes	Yes	Yes	Yes	Yes	Yes	Yes
7. Did the review authors provide a list of excluded studies and justify the exclusions?	No	Yes	No	Yes	Yes	No	No	No	No	No
8. Did the review authors describe the included studies in adequate detail?	Yes	Yes	Yes	Yes	Yes	Yes	Yes	Yes	Yes	Yes
9. Did the review authors use a satisfactory technique for assessing the risk of bias (RoB) in individual studies that were included in the review?	Yes	Yes	Yes	Yes	Yes	Yes	Yes	Yes	Yes	Yes
10. Did the review authors report on the sources of funding for the studies included in the review?	No	Yes	No	No	Yes	No	No	Yes	No	No
11. If meta-analysis was performed did the review authors use appropriate methods for statistical combination of results?	No meta-analysis	Yes	No meta-analysis	Yes	Yes	Yes	Yes	Yes	Yes	Yes
12. If meta-analysis was performed, did the review authors assess the potential impact of RoB in individual studies on the results of the meta-analysis or other evidence synthesis?	No meta-analysis	No	No meta-analysis	No	Yes	Yes	No	Yes	Yes	Yes

13. Did the review authors account for RoB in individual studies when interpreting/ discussing the results of the review?	Yes	Yes	Yes	Yes	Yes	Yes	Yes	Yes	Yes	Yes
14. Did the review authors provide a satisfactory explanation for, and discussion of, any heterogeneity observed in the results of the review?	Yes	Yes	Yes	Yes	Yes	Yes	Yes	Yes	Yes	Yes
15. If they performed quantitative synthesis did the review authors carry out an adequate investigation of publication bias (small study bias) and discuss its likely impact on the results of the review?	No meta-analysis	Yes	No meta-analysis	Yes	Yes	Yes	No	Yes	Yes	Yes
16. Did the review authors report any potential sources of conflict of interest, including any funding they received for conducting the review?	Yes	Yes	Yes	Yes	Yes	Yes	Yes	Yes	Yes	Yes
Number of critical/non-critical weaknesses	2/2	0/2	2/3	0/3	0/1	2/2	2/3	2/0	1/2	1/2
Overall confidence in results rating	Critically low	Moderate	Critically low	Moderate	High	Critically low	Critically low	Critically low	Low	Low

Yellow background, critical domain

2 systematic reviews did not carry out meta-analysis and could not be assessed on domains 11, 12 and 15. Regarding item 15, multiple studies evaluated publication bias as part of the GRADE assessment. As publication bias was assessed, they were counted as "yes", but their discussions contained little information about publication bias.

There were multiple reasons for why the quality of systematic reviews were low. There is less awareness and research on publishing high quality systematic reviews, as opposed to RCTs and observational studies. This may lead to many systematic reviews not addressing some of the critical domains mentioned. The first AMSTAR tool (48) was published in 2007, and the second updated version AMSTAR 2 was published in 2017. The changes between the two instruments may not be widely known, causing the systematic reviews to be assessed as low quality. Henschke et al (37) is a Cochrane review and scored moderate confidence, as it was first published in 2010, which was before AMSTAR 2 was published.

Discussion

Summary of results

Both standalone CBT and CBT with active treatment are more effective in reducing pain and improving disability compared to WL/UC. The evidence surrounding standalone CBT vs AT is varied, where a few systematic reviews support CBT, however it is not enough to be considered a significant effect. CBT is more effective compared to physiotherapy, operant therapy and cognitive therapy, but less effective than surgery and respondent therapy.

CBT in combination with AT is more effective than AT itself. Specific treatments such as GA, CFT, MBI with CBT have limited evidence with only one systematic review each.

Overall completeness and applicability

Not all systematic reviews measured disability as an outcome. Specific forms of CBT such as CFT, GA and MBI with CBT only had one systematic review. ACT and MBCT are some of the "third wave" behavioural therapy that could be included, however there is only 1 RCT by Godfrey et al. (49) involving ACT, and only 1 RCT related to MBCT by Day et al. (50). All the primary studies included were RCTs, which are the gold standard for clinical trials. However, observational studies could be incorporated to provide a complete picture of effects.

There is only 1 RCT conducted in 1997 by Rose et al. (46) that compared intensity, group and individual treatment. Further research is required in order to give recommendations for the most effective way of delivering CBT.

The systematic reviews include male and female genders, and age ranges between 18 to 65. However, the studies do not reflect how treatments affect people aged above 65 and populations from lower middle-income countries, as most of the studies are from higher income countries.

Potential bias

There were multiple sources of bias in this overview of reviews. Grey literature was not searched, observational studies were not included and only full texts of English papers were incorporated, leading to publication bias and language bias. A few systematic reviews that were included also reported having sources of funding or conflicts of interests, as mentioned in table 2.

Measurement bias is challenging to avoid as it arises from how pain is generally subjective, and there are no objective measurements of pain.

75% of systematic reviews in CBT + AT vs AT comparison had physiotherapy as AT, which could introduce bias as this would not be a good representation of all active treatments. When carrying out data synthesis, higher weighting could be applied for systematic reviews that have greater sample size and higher quality evidence. Ho et al. (38) had a large sample size of studies and low risk of bias, so should not be of equal weighting to other systematic reviews. Similarly, the overlap of primary studies in data synthesis should be considered. In the comparison for CBT + exercise vs surgery, Henschke et al. (37) uses the same RCTs as Rihn (41) and therefore the weighting of these studies should be lower.

Agreements and disagreements in relation to other research

As of the time of writing this overview, there is no published overview of reviews regarding CBT in CNSLBP. The results of systematic reviews that examined CBT as an adjunct therapy or comparing CBT to WL/UC demonstrated a significant effect in reducing pain and disability in CNSLBP, as per Mindouri et al (16).

The comparison CBT vs AT had differing directions of effect, this may be because the AT that were included were different treatments altogether.

Strengths

A thorough methodology was outlined and executed. The additional consideration of overlapping primary studies and qualitative data synthesis were performed via methods supported by literature on epidemiological research. There was comprehensive data extraction of each individual systematic review. Results of both individual systematic reviews and data synthesis grouped by interventions were reported, adding to our current understanding on the effect of CBT on CNSLBP.

Limitations

In this data synthesis, active treatment contains a mixture of treatments. There could have been further separation of active treatment into specific treatments to obtain more accurate findings about the efficacy of treatments compared to CBT. A meta-analysis would provide more precise information, including effect size, confidence intervals and statistics for heterogeneity.

There was high heterogeneity in study population. Some primary studies had a small sample size, leading to inadequate power of studies. Other systematic reviews have also commented on the lack of standardized outcome measures and different units of measurement that could alter the results (34, 39, 40, 43).

Although the Cochrane Handbook states to use GRADE tool to assess certainty of evidence, there have been conflicts in literature regarding this as there has been no formal guidance on using GRADE in the context of Overview of reviews (51). As such, the individual GRADE assessments from each systematic review were extracted as an alternative to conducting GRADE assessments.

Conclusions

An overview of reviews was conducted to examine how CBT affects pain intensity and disability in CNSLBP. Systematic reviews suggest that CBT is most effective when integrated with other forms of active treatment, such as exercise or physiotherapy.

Implications for current clinical practice would be to continue providing treatment programs that incorporate both physical and cognitive behavioural components. This would not only improve patient outcomes, but also restore a healthy workforce, and hence improve the economy. The availability of pain treatment programs is often region specific, with variable length in waiting times (52). The next step would be to evaluate the cost-effectiveness and make these programs more widely available.

Currently there is no agreed standard to the quality of care provided (52). Future research may need to consider the optimal duration, intensity, and mode of delivery of CBT, including group, individual, telephone or internet-based treatment. The previous study by Rose et al. (46) comparing intensity, group and individual treatment was conducted in 1997 and there could be changes in how CBT is delivered since then.

There were also specific types of CBT that were not evaluated in this overview such as ACT, which would again require further research in determining the efficacy of treatment. Future research would also be needed to standardize outcome measures to facilitate more effective comparisons across multiple studies.

References

1. Krismer M, van Tulder MW. Low back pain (non-specific). Best Practice & Research Clinical Rheumatology. 2006;21(1):77-91.
2. Koes B, van Tulder MW, Thomas S. Diagnosis and treatment of low back pain. BMJ. 2006;332:1430-4.
3. WHO. Low Back Pain: WHO; 2023 [updated 19/06/2023. Available from: https://www.who.int/news-room/fact-sheets/detail/low-back-pain.
4. Vitoula K, Venneri A, Varrassi G, Paladini A, Sykioti P, Adewusi J, et al. Behavioral Therapy Approaches for the Management of Low Back Pain: An Up-To-Date Systematic Review. Pain Ther. 2018;7(1):1-12.
5. Nordin M, Balague F, Cedraschi C. Nonspecific Lower-back Pain: Surgical versus Nonsurgical Treatment. CLINICAL ORTHOPAEDICS AND RELATED RESEARCH. 2006;443:156-67.
6. Macfarlane GJ, Beasley M, Jones EA, Prescott GJ, Docking R, Keeley P, et al. The prevalence and management of low back pain across adulthood: Results from a population-based cross-sectional study (the MUSICIAN study). PAIN. 2012;153:27-32.
7. Statistics OfN. Half a million more people are out of the labour force because of long-term sickness 2022 [Available from: https://www.ons.gov.uk/employmentandlabourmarket/peoplenotinwork/economicinactivity/articles/halfamillionmorepeopleareoutofthelabourforcebecauseoflongtermsickness/2022-11-10.
8. Maniadakis N, Gray A. The economic burden of back pain in the UK. PAIN. 2000;84:95-103.
9. NICE. Low back pain and sciatica in over 16s: assessment and management. 2016 11/12/2020.
10. Carter GT, Duong V, Ho S, Ngo KC, Greer CL, Weeks DL. Side effects of commonly prescribed analgesic medications. Physical Medicine and Rehabilitation Clinics of North America. 2014;25(2):457-70.
11. Dharap SB, Barbaniya P, Navgale S. Incidence and Risk Factors of Postoperative Complications in General Surgery Patients. Cureus. 2022;14(11).
12. Feldmann M, Hein HJ, Voderholzer U, Doerr R, Hoff T, Langs G, et al. Cognitive Change and Relaxation as Key Mechanisms of Treatment Outcome in Chronic Pain: Evidence From Routine Care. Frontiers in Psychiatry. 2021;12.
13. Sturgeon JA. Psychological therapies for the management of chronic pain. Psychology Research and Behaviour Management. 2014;7:115-24.
14. Georgescu R, Dobrean A, Predescu E. Benefits of cognitive restructuring, acceptance and distraction for pain intensity and pain tolerance. Journal of Evidence-Based Psychotherapies. 2018;18(2):143-60.
15. Hayes SC, Hofmann SG. The third wave of cognitive behavioral therapy and the rise of process-based care. World Psychiatry. 2017;16(3):245-6.
16. Mindouri A, Trevlaki E, Trevlakis E, Mallios N, Domatas G, Xristara-Papadopoulou A. The Effectiveness of Cognitive Behavioral Therapy in Chronic Low Back Pain. Biomed J Sci & Tech Res. 2023;48(3):39646-55.
17. Zhang Q, Jiang S, Young L, Li F. The Effectiveness of Group-Based Physiotherapy-Led Behavioral Psychological Interventions on Adults With Chronic Low Back Pain: A Systematic Review and Meta-Analysis. Am J Phys Med Rehabil. 2019;98(3):215-25.
18. Pollock M, Fernandes R, Becker LA, Pieper D, Hartling L. Chapter V: Overviews of Reviews. 2023. In: Cochrane Handbook for Systematic Reviews of Interventions version 64 [Internet]. Available from: https://training.cochrane.org/handbook/current/chapter-v.

19. Higgins JPT, Thomas J, Chandler J, Cumpston M, Li T, Page MJ, et al. Cochrane Handbook for Systematic Reviews of Interventions: Cochrane; 2022 [updated February 2022. Available from: www.training.cochrane.org/handbook.

20. Campbell M, McKenzie J, Sowden A, Katikireddi SV, Brennan SE, Ellis S, et al. Synthesis without meta-analysis (SWiM) in systematic reviews: reporting guideline. BMJ. 2020;368(6890).

21. Page MJ, McKenzie JE, Bossuyt PM, Boutron I, Hoffman TC, Mulrow CD, et al. The PRISMA 2020 statement: an updated guideline for reporting systematic reviews. BMJ. 2021;372.

22. Treede R, Rief W, Barke A, Aziz Q, Bennett M, Benoliel R, et al. Chronic pain as a symptom or a disease: the IASP Classification of Chronic Pain for the International Classification of Diseases (ICD-11). PAIN. 2019;160(1):19-27.

23. The EndNote Team. EndNote. EndNote 20 ed. Philadelphia, PA: Clarivate; 2013.

24. Falconer J. Removing duplicates from an EndNote library 2018 [Available from: https://blogs.lshtm.ac.uk/library/2018/12/07/removing-duplicates-from-an-endnote-library/.

25. Ouzzani M, Hammady H, Fedorowicz Z, Elmagarmid A. Rayyan—a web and mobile app for systematic reviews. Syst. 2016;5.

26. Pollock A, Fernandes RM, Newton AS, Scott SD, Hartling L. A decision tool to help researchers make decisions about including systematic reviews in overviews of reviews of healthcare interventions. Syst. 2019;8.

27. Pieper D, Antoine SL, Mathes T, Neugebauer EA, Eikermann M. Systematic review finds overlapping reviews were not mentioned in every other overview. Journal of Clinical Epidemiology. 2014;67:368-75.

28. Hennessy EA, Johnson BT. Examining Overlap of Included Studies in Meta-Reviews: Guidance for using the Corrected Covered Area Index. Research Synthesis Methods. 2020;11(1):134-45.

29. Shea BJ, Reeves BC, Wells G, Thuku M, Hamel C, Moran J, et al. AMSTAR 2: a critical appraisal tool for systematic reviews that include randomised or non-randomised studies of healthcare interventions, or both. BMJ. 2017;358.

30. Boon MH, Thomson H. The effect direction plot revisited: Application of the 2019 Cochrane Handbook guidance on alternative synthesis methods. Research Synthesis Methods. 2021;12:29-33.

31. Du S, Dong J, Jin S, Zhang H, Zhang Y. Acceptance and Commitment Therapy for chronic pain on functioning: A systematic review of randomized controlled trials. Neuroscience and Biobehavioral Reviews. 2021;131:59-76.

32. Pardos-Gascon EM, Narambuena L, Leal-Costa C, van-der Hofstadt-Roman CJ. Differential efficacy between cognitive-behavioral therapy and mindfulness-based therapies for chronic pain: Systematic review. International Journal of Clinical and Health Psychology. 2021;21(1):100197.

33. van Middelkoop M, Rubinstein SM, Kuijpers T, Verhagen AP, Ostelo R, Koes BW, et al. A systematic review on the effectiveness of physical and rehabilitation interventions for chronic non-specific low back pain. Eur Spine J. 2011;20(1):19-39.

34. Bahnamiri FA, Norouzi A, Reskati MH, Hosseini SH. Effectiveness of Mindfulness-Based Interventions on Pain Intensity in Patients with Chronic Low Back Pain: A Systematic Review. Iranian Journal of Psychiatry and Behavioral Sciences. 2020;14.

35. Devonshire JJ, Wewege MA, Hansford HJ, Odemis HA, Wand BM, Jones MD, et al. Effectiveness of Cognitive Functional Therapy for Reducing Pain and Disability in Chronic Low Back Pain: A Systematic Review and Meta-analysis. The journal of orthopaedic and sports physical therapy. 2023;0(5):1-42.

36. Hajihasani A, Rouhani M, Salavati M, Hedayati R, Kahlaee AH. The Influence of Cognitive Behavioral Therapy on Pain, Quality of Life, and Depression in Patients Receiving Physical Therapy for Chronic Low Back Pain: A Systematic Review. PM&R Journal. 2019;11(2):167-76.

37. Henschke N, Ostelo RW, van Tulder MW, Vlaeyen JW, Morley S, Assendelft WJ, et al. Behavioural treatment for chronic low-back pain. Cochrane Database of Systematic Reviews. 2010;2010(7):CD002014.
38. Ho EK, Chen L, Simic M, Ashton-James CE, Comachio J, Wang DXM, et al. Psychological interventions for chronic, non-specific low back pain: systematic review with network meta-analysis. BMJ. 2022;376:e067718.
39. Lopez-de-Uralde-Villanueva I, Munoz-Garcia D, Gil-Martinez A, Pardo-Montero J, Munoz-Plata R, Angulo-Diaz-Parreno S, et al. A Systematic Review and Meta-Analysis on the Effectiveness of Graded Activity and Graded Exposure for Chronic Nonspecific Low Back Pain. Pain Med. 2016;17(1):172-88.
40. Petrucci G, Papalia GF, Russo F, Vadala G, Piredda M, De Marinis MG, et al. Psychological Approaches for the Integrative Care of Chronic Low Back Pain: A Systematic Review and Metanalysis. International Journal of Environmental Research and Public Health. 2021;19(1):22.
41. Rihn JA, Radcliff K, Norvell DC, Eastlack R, Phillips FM, Berland D, et al. Comparative Effectiveness of Treatments for Chronic Low Back Pain: A Multiple Treatment Comparison Analysis. Clinical Spine Surgery. 2017;30(5):204-25.
42. Yang J, Lo WLA, Zheng F, Cheng X, Yu Q, Wang C. Evaluation of Cognitive Behavioral Therapy on Improving Pain, Fear Avoidance, and Self-Efficacy in Patients with Chronic Low Back Pain: A Systematic Review and Meta-Analysis. Pain Research and Management. 2022;2022:4276175.
43. Jurak I, Delas K, Erjavec L, Stare J, Locatelli I. Effects of Multidisciplinary Biopsychosocial Rehabilitation on Short-Term Pain and Disability in Chronic Low Back Pain: A Systematic Review with Network Meta-Analysis. J. 2023;12.
44. Sterne JAC, Savović J, Page MJ, Elbers RG, Blencowe NS, Boutron I, et al. RoB 2: a revised tool for assessing risk of bias in randomised trials. BMJ. 2019;366(l4898).
45. Furlan AD, Malmivaara A, Chou R, Maher CG, Deyo RA, Schoene M, et al. 2015 Updated Method Guideline for Systematic Reviews in the Cochrane Back and Neck Group. Spine. 2015;40(21):1660–73.
46. Rose MJ, Reilly JP, Pennie B, Bowen-Jones K, Stanley IM, Slade PD. Chronic low back pain rehabilitation programs: a study of the optimum duration of treatment and a comparison of group and individual therapy. Spine. 1997;22(19):2246-51.
47. Guyatt GH, Oxman AD, Vist GE, Kunz R, Falck-Ytter Y, Alonso-Coello P, et al. GRADE: an emerging consensus on rating quality of evidence and strength of recommendations. BMJ. 2008;336(7650):924-6.
48. Shea BJ, Grimshaw JM, Wells GA, Boers M, Andersson N, Hamel C, et al. Development of AMSTAR: a measurement tool to assess the methodological quality of systematic reviews. BMC Medical Research Methodology. 2007;7(10).
49. Godfrey E, Wileman V, Holmes MG, McCracken LM, Norton S, Moss-Morris R, et al. Physical Therapy Informed by Acceptance and Commitment Therapy (PACT) Versus Usual Care Physical Therapy for Adults With Chronic Low Back Pain: A Randomized Controlled Trial. The Journal of Pain. 2020;21(1-2):71-81.
50. Day MA, Ward LC, Ehde DM, Thorn BE, Burns J, Barnier A, et al. A Pilot Randomized Controlled Trial Comparing Mindfulness Meditation, Cognitive Therapy, and Mindfulness-Based Cognitive Therapy for Chronic Low Back Pain. Pain Med. 2019;20(11):2134–48.
51. Gates M, Gates A, Guitard S, Pollock M, Hartling L. Guidance for overviews of reviews continues to accumulate, but important challenges remain: a scoping review. Syst. 2020;9(254).
52. Price C, Hoggart B, Olukoga O, Williams A, Bottle A. National Pain Audit Final Report. U.K.: British Pain Society; 2012.

Supporting Information

S1 Checklist. PRISMA Checklist.

S2 Checklist. PRISMA Abstract Checklist.

S3 Supporting Information. Search Strategy.

S4 Table. Citation Matrix of Primary Studies.

S5 Table. Results of Included Systematic Reviews.

S6 Table. Systematic reviews involved in each comparison.

Fig 1. PRISMA flow diagram

Click here to access/download;Figure;Fig 1. PRISMA flow diagram.docx

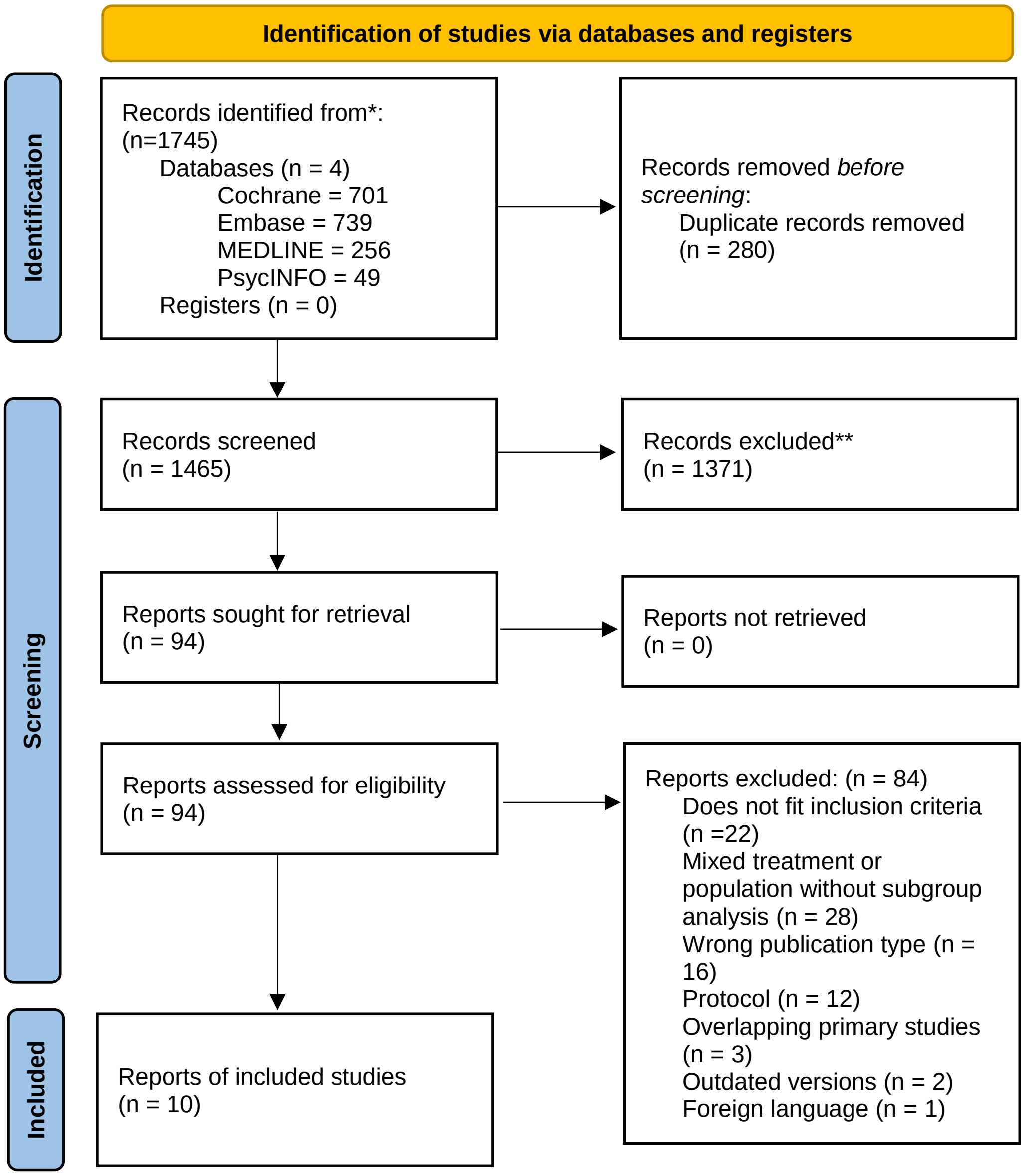

Click here to access/download
Supporting Information
S1 Checklist. PRISMA Checklist..docx

Click here to access/download
Supporting Information
S2 Checklist. PRISMA Abstract Checklist..docx

Click here to access/download
Supporting Information
S3 Supporting Information. Search Strategy..docx

Click here to access/download
Supporting Information
S4 Table. Citation Matrix of Primary Studies..docx

S5 Table. Results of Included Systematic Reviews

Click here to access/download
Supporting Information
S5 Table. Results of Included Systematic Reviews..docx

S6 Table. Systematic reviews involved in each comparison

Click here to access/download
Supporting Information
S6 Table. Systematic reviews involved in each comparison..docx
